# Unveiling the *Allium ampeloprasum* rhizosphere microbiome and its functional dataset under different fertilization systems

**DOI:** 10.1186/s12863-025-01349-8

**Published:** 2025-08-06

**Authors:** Olubukola Oluranti Babalola, Oluwaseun Emmanuel Shittu, Ben Jesuorsemwen Enagbonma

**Affiliations:** 1 Food Security and Safety Focus Area, Faculty of Natural and Agricultural Sciences, North-West University, Private Mail Bag X2046, Mmabatho, 2735 South Africa; 2 Department of Life Sciences, Imperial College London, Silwood Park Campus, Buckhurst Road, Ascot, Berkshire, SL5 7PY UK

**Keywords:** Food security, Leek, Functionality, Metabolism, Biofertiliser, Chemical fertiliser, Shotgun sequencing

## Abstract

Leek (*Allium ampeloprasum*) is a nutritious vegetable popularly cultivated in South Africa and most regions of the world. It is generally recognised as a source of vitamins and vegetables. Nevertheless, little is known about its rhizosphere microbiome and its microbial functional dataset under various fertilization systems. Therefore, this study intended to unveil the rhizosphere microbiome of *Allium ampeloprasum* and their functional datasets through shotgun metagenomics sequencing analysis.

## Data description

Soil samples were collected from three different plots; chemical fertilizer (G1), biofertilizer plot (G2) and Bulk soil (G3) plots with each plot consisting of four replicates. The entire DNA was mined from soil samples with DNeasy^®^ PowerSoil pro kit and shotgun metagenomic technique was then used to sequence the extracted DNA. The datasets revealed that biofertilizer enriched the rhizosphere microbiomes and their functional categories, and were predominated by bacteria at the kingdom level while metabolism dominated the functional categories (Table 1, Data file 5). Information on the clean metagenomic datasets is deposited in NCBI-SRA with accession numbers SRP537120, SRP537121, and SRP537188 (Table 1; Data files 1, 2, and 3). By providing insights into microbial shifts and their functions driven by different fertilisation systems, these findings imply that biofertilizer can enhance soil health, leading to more productive and sustainable agricultural practices.

**Table 1:** An overview of the data sets or files

Label	ID of data set/data file	File extension (Type of files)	Data source and identification (access number or DOI)
Data file 1	Genome sequence data of *Allium ampeloprasum* rhizosphere soil from the biofertiliser plot.	FASTQ file (.fastq)	NCBI Sequence Read Archive (http://identifiers.org/insdc.sra:SRP537120) [17].
Data file 2	Genome sequence data of *Allium ampeloprasum* rhizosphere soil samples from the chemical fertiliser plot.	FASTQ file (.fastq)	NCBI Sequence Read Archive (http://identifiers.org/insdc.sra:SRP537121) [18].
Data file 3	Genome sequence data of uncultivated bulk soil.	FASTQ file (.fastq)	NCBI Sequence Read Archive (http://identifiers.org/insdc.sra:SRP537188) [19].
Data file 4	Nucleic acid information	Word document files (.docx)	Figshare (10.6084/m9.figshare.28925831) [20].
Data file 5	Major microbiome and functional distributions	Word document files (.docx)	Figshare (10.6084/m9.figshare.28930460) [21].

### Objectives

*Allium ampeloprasum L*. is known to be classified under the genus *Allium* (Alliaceae) [1], which is widely distributed and consumed globally. Allium families are recognised to be a prodigious home of various vitamins, including vitamin A, C, and K, as well as a number of B vitamins, including B6 and folate. These plants also contain antioxidant properties and are high in dietary fibre when consumed as vegetables, making them important in food nutrition [2]. The viable host of microorganisms in their rhizosphere could actively impact the physiological growth and development of *Allium ampeloprasum* plants. Understanding the community structure of its rhizosphere microbiome and functional categories of agricultural importance under different fertilisation will help in agricultural sustainability. Profiling the community organization and microbiome diversity related to *Allium ampeloprasum’s* rhizosphere under different fertilisation systems using a shotgun sequencing approach is essential in comprehending the complex impacts of agro-chemical fertilisers vs. biofertiliser on microbial ecosystems, which carry out an essential functions in plant health, sustainable agriculture and soil fertility. Among the various fertilisation methods utilised in agriculture, the treatment of plants with organic fertilisers (biofertilisers) is recognised for enhancing and boosting the diversity of microorganisms and the community structure within agricultural lands [3]. In this context, shotgun sequencing as used in this study offers a significant advantage over traditional microscopic techniques and 16 S rRNA sequencing, providing a detailed and comprehensive analysis of communities of microorganisms by capturing the full spectrum of microbial life, including archaea, fungi, bacteria and viruses, without being limited to specific regions of the genome [4]. Hence, this study profiled the functional and structural diversity of the microbiome from the rhizosphere of *Allium ampeloprasum* under different fertilisation systems.

### Description of data

The soil samples utilised for the whole genome sequencing were sourced from Rosaly farm, located in the Gauteng province of South Africa. The farm covers an area of 15,000 m^2^ and is divided into three plots treated with chemical fertiliser (G1), biofertilizer (G2) and uncultivated bulk soil (G3), with each plot at a distance of 20 meters apart from the next. The sampling sites (latitude: S 26^o^6’10.72764”, longitude: E 27^o^34’41.7576) have an average temperature of 22 °C, and yearly rainfall of 794 mm. Soil samples were independently sourced from the rhizosphere of *Allium ampeloprasum* crop (15 cm depth) in fertilisation plots during the flowering or bolting stage, on day 57 after sowing, and from uncultivated bulk soil. Within each plot, four replicate samples were taken: L1, L2, L3 and L4 for (G1); L9, L10, L11 and L12 for (G2); and LB1, LB2, LB3 and LB4 for (G3), resulting in a total of 12 samples. Plot A has a history of chemical fertiliser application, consisting of calcium, magnesium and potassium nitrate; ammonium and potassium sulphate; and N.P.K. (5:1:5). Plot B with organic fertilisers like Terramax (PGPRs), Humesoil (plant-derived materials), and Soluphos (PSM), while the bulk soil samples were taken from an uncultivated plot, 20 m away from fertilised plots, to prevent contamination from fertilised treatments. The soil samples were conveyed into the laboratory in an ice box and preserved at 4 °C.

20 g samples from each plot [5] were employed for the extraction of DNA through the PowerSoil DNeasy^®^ pro kit. The DNA extracted were then sequenced via the Illumina NovaSeq X Plus (PE 150) platform (Table 1, Data file 4) [3]. A Covaris disruptor was used to fragment the DNA to about 350 bp, then PCR amplification, adapter ligation and end repair were performed to prepare the library. AATI analysis was used to confirm qPCR validated concentrations greater than 3 nM and the library’s insert size. Using PE150 technology, quality-assured libraries were combined and sequenced. Preprocessing of data was carried out using Fastp [6] to eliminate poor-quality readings and adapters, and Bowtie2 [7] was utilised to remove impurities from the host. With the help of the meta-large preset MEGAHIT, the clean reads were put together [8], and ‘N’ junction scaffolds were thrown away in order to create scaftigs [9]. MetaGeneMark was used for ORF prediction [10]. A non-redundant gene catalog was produced by using CD-HIT to eliminate redundant ORFs [11]. Using the blastp algorithm (DIAMOND), the unigenes were aligned to the Micro_NR database as described by B Buchfink, C Xie and DH Huson [12], using 1e-5 as e-value cutoff [13]. LCA algorithm was employed for species annotations assignment in MEGAN software [14]. Relative abundances were determined using the R ade4 software [15]. Diversity indices were computed with “vegan” R software [16], with ANOSIM used to evaluate group differences.

## Data Availability

This data note provides open access to all the associated datasets via the NCBI Sequence Read Archive SRP537121 (Data file 1), SRP537120 (Data file 2), and SRP537188 (Data file 3) and Figshare. http://identifiers.org/insdc.sra: SRP537121 (Data file 1), http://identifiers.org/insdc.sra: SRP537120 (Data file 2), http://identifiers.org/insdc.sra: SRP537188 (Data file 3), https://doi.org/10.6084/m9.figshare.28925831 (Data file 4), and https://doi.org/10.6084/m9.figshare.28930460 (Data file 5). Details and links to the data can be found in Table 1 and references (Babalola et al., 2025a, 2025b; NCBI Sequence Read Archive, 2024; NCBI Sequence Read Archive, 2024a, 2024b).

## Abbreviations

L1, L2, L3 and L4: Are replicated soil samples from *Allium ampeloprasum* rhizosphere under chemical fertilisation
L9, L10, L11, and L12: Are replicated soil samples from the *Allium ampeloprasum* rhizosphere under biofertiliser application
LB1, LB2, LB3, and LB4: Are replicated soil samples from bulk soil
PGPR: Plant growth-promoting rhizobacteria
